# Supplementary material for: Exploring the hidden interior of the Earth with directional neutrino measurements
Source: Nat Commun. 2017 Jul 10;8:15989. doi: 10.1038/ncomms15989 (PMC5508127; doi:10.1038/ncomms15989)
Supplement: Supplementary Information [file ncomms15989-s1.pdf]

Type of file: PDF
Title of file for HTML: Supplementary Information
Description: Supplementary Figures, Supplementary Tables, Supplementary Notes and Supplementary References.

Type of file: PDF
Title of file for HTML: Peer Review File
Description:

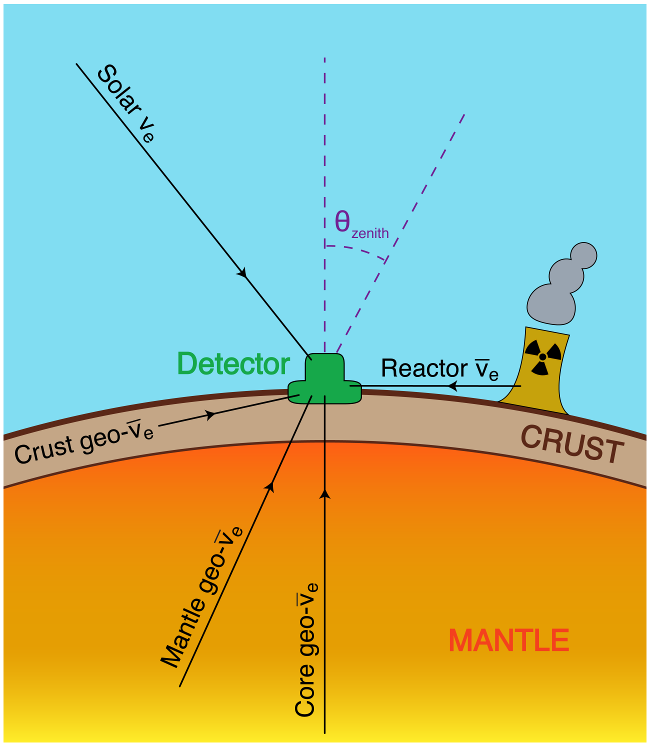

Supplementary Figure 1: **Schematic of signal and background neutrino sources.** Neutrino sources considered in this paper include geo-neutrinos (geo-$\overline{\nu}_e$) from the Earth's crust, mantle and core; solar neutrinos (solar ν_e) from the Sun; and reactor anti-neutrinos (reactor $\overline{\nu}_e$) from man-made nuclear reactors. θ_{zenith} is the angle measured from the vertical axis, where the vertical axis is defined opposite the direction pointing to the center of the Earth. Figure is not drawn to scale.

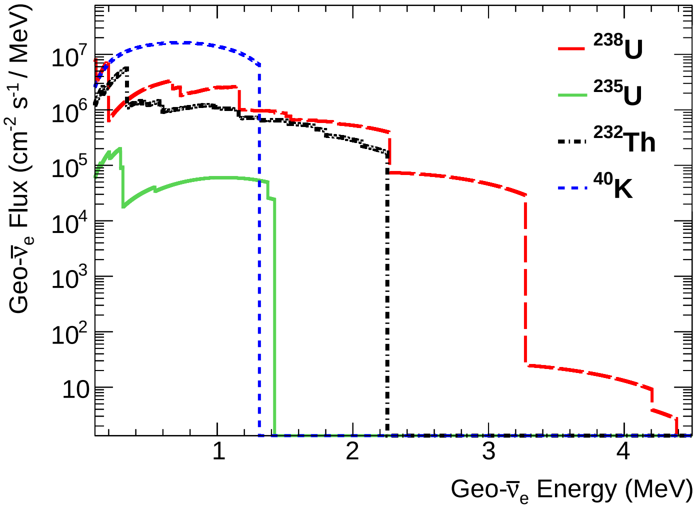

Supplementary Figure 2: **Predicted flux of geo-neutrinos at Kamioka.** The predicted geo-neutrino flux at Kamioka (Japan) is shown here for decays of ^{238}U (red long-dashed), ^{235}U (green solid), ^{232}Th (black dash-dotted) and ^{40}K (blue short-dashed) as a function of neutrino energy (MeV), not including the effect of neutrino oscillation. Flux spectra are taken from Enomoto[1], with normalization according to Table 1.

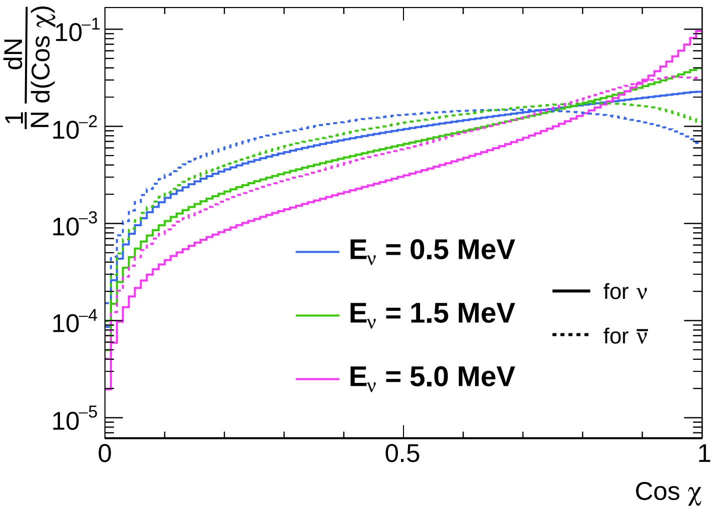

Supplementary Figure 3: **Differential neutrino-electron elastic scattering cross section.** Cross sections are shown here versus scattering angle (χ) for three representative neutrino energies, E_ν = 0.5, 1.5 and 5.0 MeV, integrated over the allowed kinematic range of electron recoil kinetic energies, $0 < T < T_{\max}$. Solid (dashed) lines represent incident neutrinos (anti-neutrinos). Each curve has been normalized to unit area.

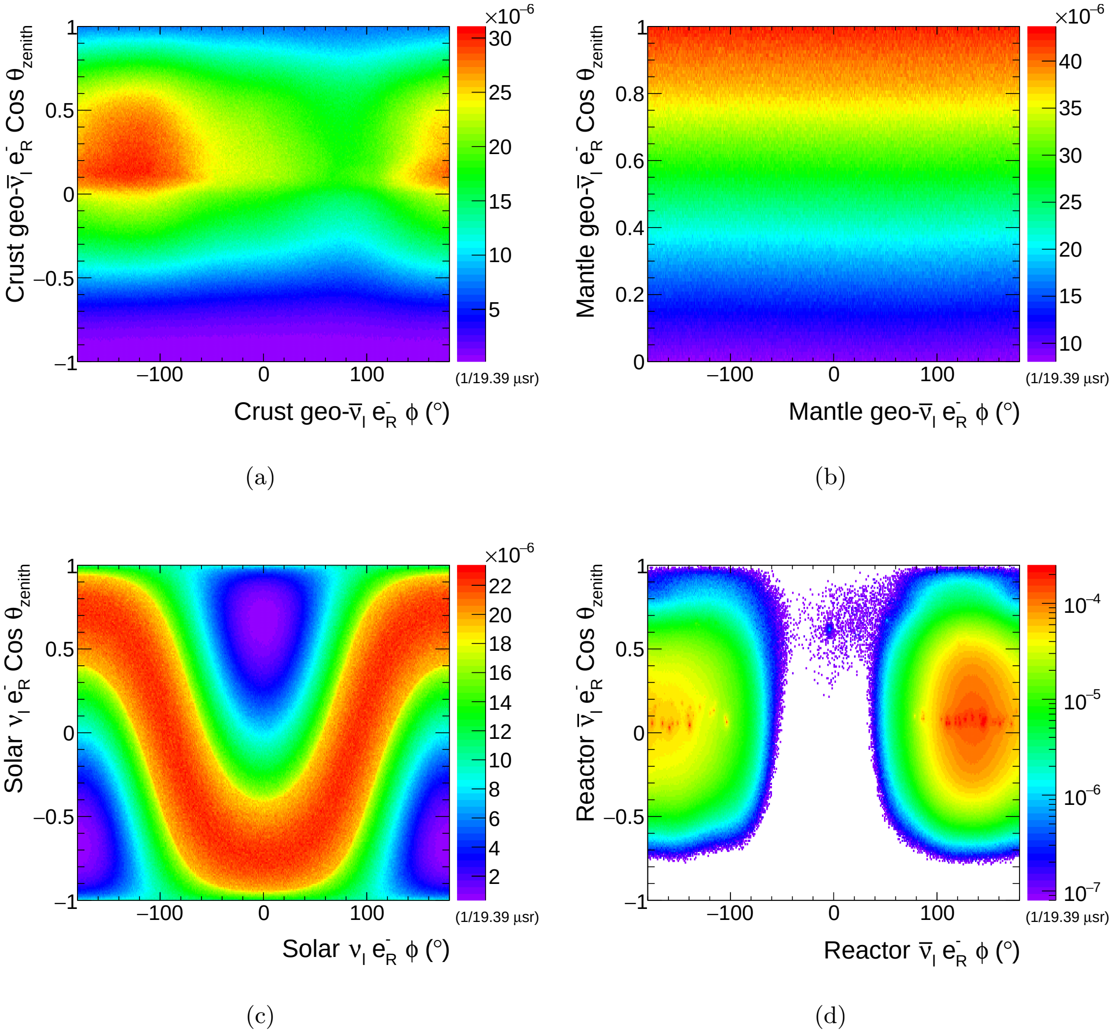

Supplementary Figure 4: **Angular distribution of electron recoils induced by neutrinos.** Angular distributions are calculated at Gran Sasso (Italy) and shown here for electron recoils induced by neutrinos from **(a)** the Earth's crust, **(b)** the Earth's mantle, **(c)** the Sun, and **(d)** worldwide nuclear reactors. The azimuthal angle ϕ is measured clockwise from due North, while the zenith angle θ_{zenith} is measured with respect to the vertical axis, defined opposite the direction pointing to the center of the Earth. Reconstructed angles have been smeared with a Gaussian distribution according to the angular resolution parameterization given in equation (1). An electron energy threshold of 250 keV has been applied to all distributions. All plots are normalized to unit volume.

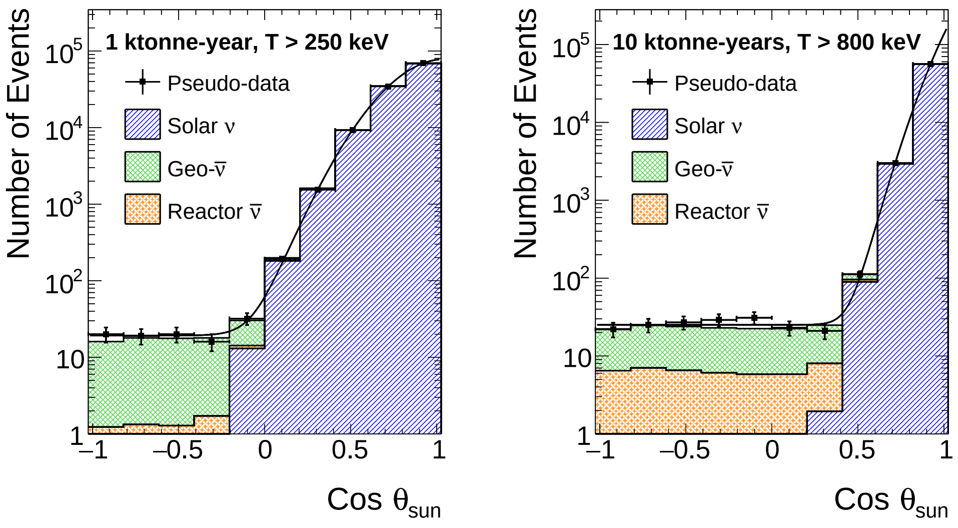

(a) (b)

Supplementary Figure 5: **Solar angular separation of electron recoils induced by neutrinos.** Angular separation from the Sun ($\theta_{\rm sun}$) of electron recoils induced by solar neutrinos (blue striped), geo-neutrinos (green hatched), and reactor anti-neutrinos (orange weave), calculated at Gran Sasso (Italy). Pseudo-experimental data (black squares) are also shown, with their statistical errors. Distributions are normalized to an exposure of **(a)** 1 ktonne-year or **(b)** 10 ktonne-years, including the effect of neutrino oscillation, with an applied electron energy threshold of $T >$ **(a)** 250 keV or **(b)** 800 keV. Pseudo-experimental data is fit using the functional form from equation (9), with $\chi^2/N_{\rm dof} =$ **(a)** 6.64/6 and **(b)** 2.16/6. Reconstructed angles have been smeared with a Gaussian distribution according to the angular resolution parameterization given in equation (1).

Source	Predicted Φ ($cm^{-2}\,s^{-1}$)	Measured Φ ($cm^{-2}\,s^{-1}$)	Energy (MeV)
pp	$5.97^{+0.04}_{-0.03} \times 10^{10}$	$6.6^{+0.7}_{-0.7} \times 10^{10}$	< 0.4
^{7}Be	$4.80^{+0.24}_{-0.22} \times 10^{9}$	$4.75^{+0.26}_{-0.22} \times 10^{9}$	0.3, 0.8
^{13}N	$5.0^{+8.6}_{-3.0} \times 10^{8}$	$< 7.7 \times 10^{8}$	< 2
^{15}O	$1.3^{+1.3}_{-0.9} \times 10^{8}$	$< 7.7 \times 10^{8}$	< 2
^{17}F	$5.52^{+79.48}_{-5.52} \times 10^{6}$	–	< 2
^{8}B	$5.16^{+0.13}_{-0.09} \times 10^{6}$	$5.02^{+0.17}_{-0.19} \times 10^{6}$	< 12
hep	$1.9^{+1.2}_{-0.9} \times 10^{4}$	–	< 18
pep	$1.45^{+0.01}_{-0.01} \times 10^{8}$	$1.6^{+0.3}_{-0.3} \times 10^{8}$	1.4
Total	$6.53^{+0.10}_{-0.05} \times 10^{10}$	–	

Supplementary Table 1: **Predicted and measured flux of solar neutrinos.** Solar neutrino flux (Φ) due to various nuclear fusion reactions, taken from the solar flux model of Bergstrom et al.[2] and measurements by the Borexino collaboration[3–5]. Values do not include the effect of neutrino oscillation. The measured proton-proton (*pp*), ^{7}Be, proton-electron-proton (*pep*), and ^{8}B contributions are all in good agreement with model predictions. The CNO flux is constrained[3] to $< 7.7\times10^{8}\,cm^{-2}\,s^{-1}$ at 95% CL, also in agreement with the solar neutrino flux model. The remaining fluxes have not yet been directly observed, but the predictions are reasonably well constrained and have uncertainties at the level of 45% (CNO) and 77% (*hep*)[6].

Site	Φ ($10^{6}\,cm^{-2}\,s^{-1}$)
Kamioka *(2010)*	3.40 ± 0.20
Kamioka	0.16 ± 0.01
Gran Sasso	0.49 ± 0.03
SNOLab	1.16 ± 0.07

Supplementary Table 2: **Predicted flux of reactor anti-neutrinos at three underground sites.** Anti-neutrino flux (Φ) from worldwide nuclear reactors, calculated at Kamioka (Japan), Gran Sasso (Italy), and SNOLab (Canada) by averaging reactor powers reported in a reference worldwide reactor model[7] over 12 calendar months from 2014 (or 2010, where noted). The 2014 (2010) case at the Kamioka site corresponds to a scenario in which all Japanese nuclear reactors have ceased (resumed) operation. Values given here do not include the effect of neutrino oscillation. A total uncertainty of 6% is estimated for all sites to account for uncertainties on the energy spectrum, reported reactor powers, and oscillation parameters (see Methods).

Source	Total	> 200 keV	> 250 keV	> 800 keV
^{238}U	61.26 ± 16.68	35.60 ± 9.69	31.73 ± 8.64	7.44 ± 2.02
^{235}U	1.36 ± 0.37	0.68 ± 0.19	0.59 ± 0.16	0.05 ± 0.01
^{232}Th	44.19 ± 9.17	24.18 ± 5.02	21.55 ± 4.47	4.65 ± 0.96
^{40}K	272.17 ± 69.90	126.66 ± 32.53	105.97 ± 27.21	4.69 ± 1.21
geo-$\overline{\nu}_\ell$ total	378.98 ± 72.44	187.12 ± 34.31	159.83 ± 28.90	16.83 ± 2.55
reactor $\overline{\nu}_\ell$	17.69 ± 1.06	13.35 ± 0.80	12.50 ± 0.75	6.42 ± 0.38

Supplementary Table 3: **Predicted number of geo-neutrino and reactor anti-neutrino events.** Event rates (per ktonne-year exposure) are calculated at Gran Sasso (Italy) and given here for three electron recoil energy thresholds, assuming a CF_4 target and 45% probability of oscillation into ν_μ or ν_τ. Rates (uncertainties) are calculated assuming the predicted flux normalizations (uncertainties) from Tables 1 and 2, respectively. Event rates are approximately 0.4% higher for a SF_6 target, or 13.8% lower for a Xe target.

Source	Total	> 200 keV	> 250 keV	> 800 keV
pp	$433.97^{+2.69}_{-2.40}$	$16.09^{+0.10}_{-0.09}$	$0.50^{+0.00}_{-0.00}$	$0.00^{+0.00}_{-0.00}$
^{7}Be (1)	$156.78^{+7.84}_{-7.19}$	$105.88^{+5.29}_{-4.85}$	$93.59^{+4.68}_{-4.29}$	$0.00^{+0.00}_{-0.00}$
^{7}Be (2)	$3.57^{+0.18}_{-0.16}$	$0.43^{+0.02}_{-0.02}$	$0.00^{+0.00}_{-0.00}$	$0.00^{+0.00}_{-0.00}$
^{13}N	$13.88^{+23.88}_{-8.33}$	$8.47^{+14.57}_{-5.08}$	$7.30^{+12.56}_{-4.38}$	$0.24^{+0.41}_{-0.14}$
^{15}O	$5.64^{+5.64}_{-3.90}$	$4.16^{+4.16}_{-2.88}$	$3.79^{+3.79}_{-2.62}$	$0.95^{+0.95}_{-0.66}$
^{17}F	$0.23^{+3.36}_{-0.23}$	$0.17^{+2.48}_{-0.17}$	$0.16^{+2.29}_{-0.16}$	$0.04^{+0.56}_{-0.04}$
^{8}B	$1.80^{+0.05}_{-0.03}$	$1.75^{+0.04}_{-0.03}$	$1.73^{+0.04}_{-0.03}$	$1.58^{+0.04}_{-0.03}$
hep	$0.01^{+0.01}_{-0.00}$	$0.01^{+0.01}_{-0.00}$	$0.01^{+0.01}_{-0.00}$	$0.01^{+0.01}_{-0.00}$
pep	$9.39^{+0.08}_{-0.08}$	$7.73^{+0.07}_{-0.07}$	$7.33^{+0.07}_{-0.07}$	$3.09^{+0.03}_{-0.03}$
solar ν_ℓ total	$625.28^{+26.12}_{-11.92}$	$144.70^{+16.24}_{-7.60}$	$114.40^{+14.12}_{-6.67}$	$5.90^{+1.18}_{-0.68}$

Supplementary Table 4: **Predicted number of solar neutrino events.** Event rates (per tonne-year exposure) are given here for three electron recoil energy thresholds, assuming a CF_4 target and 45% probability of oscillation into ν_μ or ν_τ. Rates (uncertainties) are calculated assuming the predicted flux normalizations (uncertainties) from Supplementary Table 1. Event rates are approximately 0.4% higher for a SF_6 target, or 13.8% lower for a Xe target.

Reservoir/Isotope	U (μg/g)	Th (μg/g)	K (wt. %)
Upper continental crust	2.7 ± 0.6	10.5 ± 1.1	2.3 ± 0.2
Middle continental crust	1.3 ± 0.4	6.5 ± 0.5	1.9 ± 0.3
Lower continental crust	0.20 ± 0.11	1.3 ± 0.9	0.71 ± 0.28
Continental lithospheric mantle	0.045 ± 0.035	0.24 ± 0.19	0.04 ± 0.03
Sediment	1.7 ± 0.1	8.1 ± 0.6	1.8 ± 0.1
Oceanic crust	0.07 ± 0.02	0.21 ± 0.06	0.07 ± 0.02
Mantle (no radioactivity in core)	0.011 ± 0.009	0.036 ± 0.033	0.016 ± 0.013
Mantle (10 p.p.b. U, Th in core)	0.008 ± 0.009	0.033 ± 0.033	0.011 ± 0.013
Core	0.010	0.010	0.000

Supplementary Table 5: **Element abundances in eight geochemical reservoirs.** Abundance of uranium (U), thorium (Th), and potassium (K) in eight geochemical reservoirs, assuming a sub-crustal Earth model with a homogeneous mantle and either no radioactivity or 10 p.p.b. U, Th in the core. Values given here are used to calculate the predicted geo-neutrino flux from the geophysical response (see Methods). Mantle values and uncertainties are constrained using measurements by the KamLAND[8] and Borexino[9] collaborations.

Isotope	Neutrino luminosity ($g^{-1}\,s^{-1}$)	Natural abundance (%)
^{238}U	7.46×10^4	99.27
^{235}U	3.20×10^5	0.7204
^{232}Th	1.62×10^4	100.0
^{40}K	2.31×10^5	0.0117

Supplementary Table 6: **Isotopic neutrino luminosities and natural abundances.** Neutrino luminosity ($g^{-1}\,s^{-1}$) and natural abundance (%) of uranium (U), thorium (Th), and potassium (K) radioactive isotopes. Values given here are used to calculate the predicted geo-neutrino flux from the geophysical response (see Methods).

Supplementary Note 1

Although the majority of geo-neutrinos are anti-neutrinos ($\overline{\nu}_e$), a small fraction are neutrinos (ν_e); however, we will generically refer to them as $\overline{\nu}_e$ here. For ^{40}K, the neutrino spectrum is composed of 10.7% ν_e from electron capture and 89.3% $\overline{\nu}_e$ from beta (β^-) decay.

Supplementary Note 2

Neutrinos created with a specific lepton flavor (electron, muon, or tau) can later be measured to have a different flavor—a phenomenon known as neutrino oscillation. The probability of oscillation (or survival) varies periodically as the neutrino propagates through space and depends on L/E_ν, where L is the baseline, or distance it has traveled, and E_ν is its energy. In the baseline and energy range of interest for this analysis, $500\,\mathrm{km} < L < 1\,\mathrm{A.U.}$ and $200\,\mathrm{keV} < E_\nu < 11\,\mathrm{MeV}$. L/E_ν is therefore $\gg 1/\Delta m_{12}^2$ and the survival probability after oscillation averages to $(1 - 0.5\,\sin^2\,2\theta_{12}) = 0.55$. Here, Δm_{12} is the mass difference between the two neutrino mass eigenstates and θ_{12} parameterizes the rotation between the flavor and mass bases.

Supplementary Note 3

Water Cherenkov detectors, although direction-sensitive, typically have energy thresholds too high for triggering on geo-$\overline{\nu}_e$s, while liquid scintillating detectors require further development of techniques for resolving direction.

Supplementary Note 4

Electron tracks with energies as low as 5.9 keV have been successfully reconstructed in small-scale detectors[10], with simulations showing that the reconstruction efficiency reaches ∼100% at 10 keV. Several direction-sensitive dark matter search experiments have demonstrated vector track reconstruction at low recoil energies using gas-filled TPCs at ∼0.1 bar. The DMTPC collaboration measured an angular resolution[11,12] of 40° for nuclear (F) recoils with kinetic energy above 50 keV and 15° above 100 keV in CF_4. The track-finding efficiency[13] was measured to be 95% above 80 keV. The NEWAGE collaboration has measured an angular resolution[14] of 55° for nuclear recoil tracks above 100 keV in a CF_4 gas-filled TPC with μ-PIC strip and gas electron multiplier (GEM) readout. The track detection efficiency was measured to be 80% at an energy threshold of 100 keV.

Supplementary Note 5

A spectral analysis of the electron recoil events pointing anti-parallel to the Sun ($\cos\theta_{\mathrm{sun}} < 0$) at the time of interaction should, in principle, exhibit a unique signature (namely, a kink) at 1.3 MeV due to the fall-off of the ^{40}K energy spectrum. This spectral feature can aid considerably in justifying the claim of a detected signal associated to ^{40}K geo-$\overline{\nu}_e$s.

Supplementary References

1. Enomoto, S. *Neutrino Geophysics and Observation of Geo-neutrinos at KamLAND.* Ph.D. thesis, Tohoku Univ. (2005).

2. Bergstrom, J. *et al.* Updated determination of the solar neutrino fluxes from solar neutrino data. *JHEP* **03**, 132 (2016).

3. Bellini, G. *et al.* First Evidence of *pep* Solar Neutrinos by Direct Detection in Borexino. *Phys. Rev. Lett.* **108**, 051302 (2012).

4. Bellini, G. *et al.* Final results of Borexino Phase-I on low-energy solar neutrino spectroscopy. *Phys. Rev.* **D89**, 112007 (2014).

5. Bellini, G. *et al.* Neutrinos from the primary proton–proton fusion process in the Sun. *Nature* **512**, 383–386 (2014).

6. Gonzalez-Garcia, M. C., Maltoni, M. & Salvado, J. Direct determination of the solar neutrino fluxes from solar neutrino data. *JHEP* **05**, 072 (2010).

7. Baldoncini, M. *et al.* Reference worldwide model for antineutrinos from reactors. *Phys. Rev.* **D91**, 065002 (2015).

8. Gando, A. *et al.* Reactor On-Off Antineutrino Measurement with KamLAND. *Phys. Rev.* **D88**, 033001 (2013).

9. Agostini, M. *et al.* Spectroscopy of geoneutrinos from 2056 days of Borexino data. *Phys. Rev.* **D92**, 031101 (2015).

10. Billard, J., Mayet, F. & Santos, D. Low energy electron/recoil discrimination for directional Dark Matter detection. *Journal of Cosmology and Astroparticle Physics* **2012**, 020 (2012).

11. Ahlen, S. *et al.* First Dark Matter Search Results from a Surface Run of the 10-L DMTPC Directional Dark Matter Detector. *Phys. Lett.* **B695**, 124–129 (2011).

12. Dujmic, D. *et al.* Charge amplification concepts for direction-sensitive dark matter detectors. *Astropart. Phys.* **30**, 58–64 (2008).

13. Lopez, J. P. *First results from a 20-liter prototype dark matter detector with directional sensitivity.* Ph.D. thesis, Massachusetts Institute of Technology (2014).

14. Miuchi, K. *et al.* First underground results with NEWAGE-0.3a direction-sensitive dark matter detector. *Phys. Lett.* **B686**, 11–17 (2010).
